# Safe Airway Management in a Pediatric Case of PTEN Hamartoma Tumor Syndrome With Multiple Pharyngeal and Laryngeal Polyps: A Case Report

**DOI:** 10.7759/cureus.91400

**Published:** 2025-09-01

**Authors:** Chida Rie, Morichi Shinichiro, Ishii Hiroki, Matsumoto Rika, Nishimata Shigeo, Gaku Yamanaka

**Affiliations:** 1 Department of Pediatrics, Tokyo Medical University Hachioji Medical Center, Tokyo, JPN; 2 Department of Pediatrics and Adolescent Medicine, Tokyo Medical University, Tokyo, JPN; 3 Department of Pathophysiology, Tokyo Medical University, Tokyo, JPN

**Keywords:** difficult intubation, laryngeal polyp, nasotracheal fiberoptic intubation, pten hamartoma tumor syndrome, upper airway obstruction

## Abstract

Safe airway management is essential, as even brief airway obstruction under anesthesia can lead to brain injury. Patients with PTEN hamartoma tumor syndrome (PHTS) undergo tonsillectomy more frequently than the general population. Although physical features of PHTS are associated with difficult intubation, prior literature has not addressed airway management in pediatric PHTS cases. This case highlights the importance of preoperative airway evaluation and planning to ensure safe airway management. The case was a four-year and three-month-old boy with PHTS who visited our hospital with a one-month history of cough and snoring. His tonsils were remarkably enlarged, and sleep apnea was observed. Tonsillectomy was planned, and preoperative nasopharyngolaryngoscopy revealed multiple polyps in the hypopharynx and larynx. He was previously diagnosed with difficult intubation because macrocephaly limited head movement, obstructing visualization of the glottic orifice during colorectal polypectomy. Therefore, we considered that multiple polyps in the hypopharynx and larynx could be another risk factor for difficult intubation. For safety management, fiberoptic nasotracheal intubation was selected, and airway complications were prevented. Preoperative airway assessment beyond bedside physical examination is necessary to accurately identify the risk of difficult intubation and consider safe airway management plans to avoid the worst-case scenario of airway management.

## Introduction

PTEN hamartoma tumor syndrome (PHTS) is an autosomal dominant cancer predisposition syndrome caused by mutations in the tumor suppressor gene PTEN (OMIM 601728), encompassing Cowden syndrome and Bannayan-Riley-Ruvalcaba syndrome [1]. The major clinical findings in children with PHTS include developmental delay, intellectual disability, autism, macrocephaly, and pigmented penile macules [1]. If the PTEN protein is absent, decreased, or dysfunctional, phosphorylation of AKT1 would be uninhibited, which causes the inability to activate cell cycle arrest and to undergo apoptosis. Moreover, a lack of protein phosphatase activity causes dysregulation of the mitogen-activated protein kinase pathway, which leads to abnormal cell survival [2]. These mechanisms put children with PHTS at an increased risk of developing benign and malignant tumors, including thyroid, breast, endometrium, skin, kidneys, central nervous system, and gastrointestinal tract tumors, but there is also an increased risk of developing papillary and lymphoid hyperplasia in the airway and thymus [1]. These papillomas and lymphoid lesions directly or indirectly obstruct the airway and may require surgical intervention with endotracheal intubation to maintain airway patency. To successfully perform endotracheal intubation, alignment of the oral, pharyngeal, and laryngeal axes and visibility of the pharyngeal and laryngeal structures are important [3,4]; however, PHTS can physically affect these factors, making intubation difficult.

Herein, we report a pediatric case of PHTS that required fiberoptic nasotracheal intubation with spontaneous breathing due to difficult intubation caused by macrocephaly and multiple polyps in the hypopharynx and larynx.

## Case presentation

**Figure 1:**
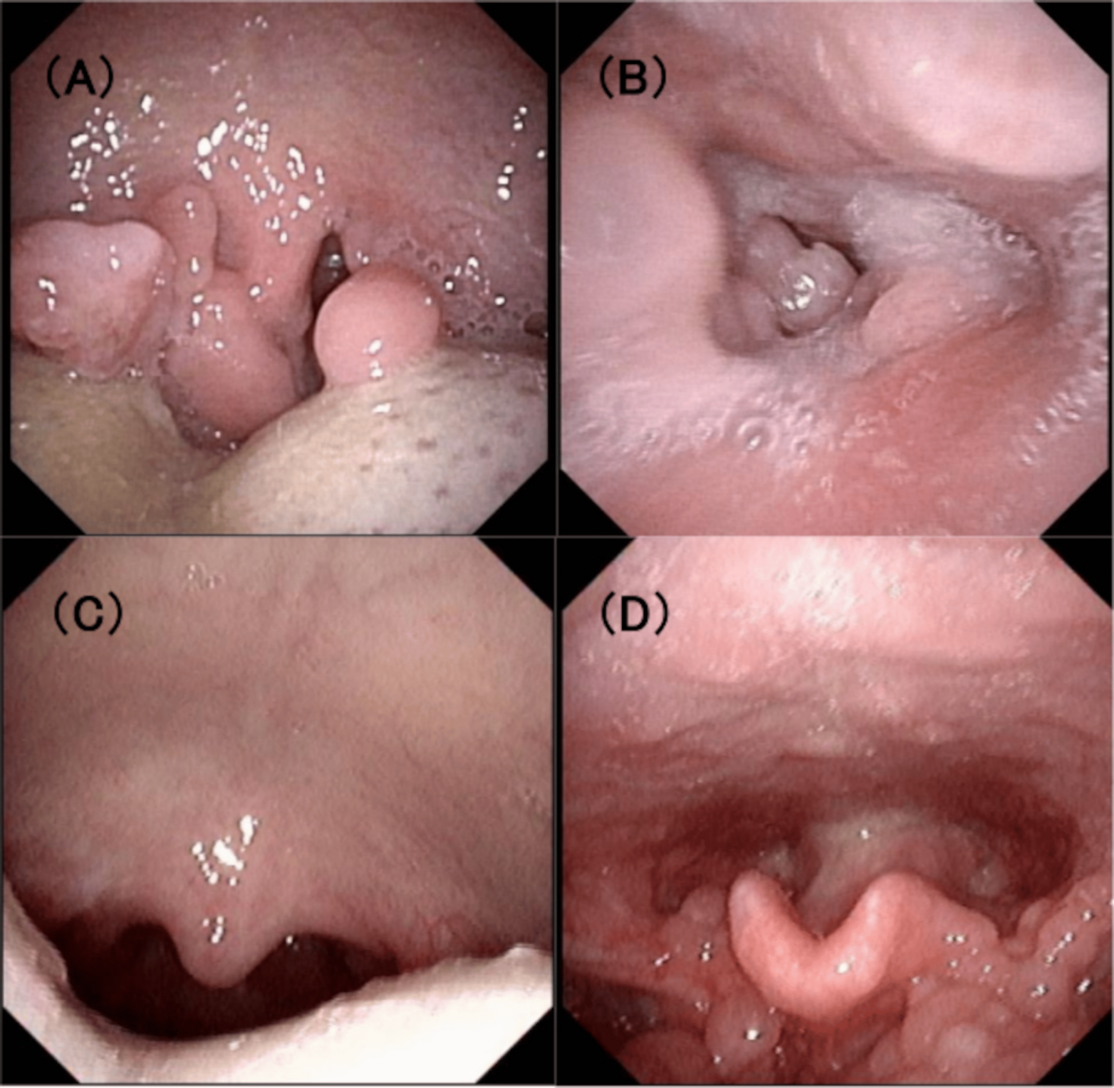
Nasopharyngolaryngoscopic findings (A) The patient at four years and three months old. Hyperplastic tonsils narrow the oral cavity. (B) The patient at four years and three months old. Multiple polyps in the hypopharynx. (C) The patient at seven years old. No relapse of tonsillar hyperplasia after tonsillectomy. (D) The patient at seven years old. Nonremarkable changes in laryngeal polyps after three years of observation.

A two-month-old boy was referred to our hospital for evaluation of macrocephaly (head circumference, 43 cm;＋4.4 standard deviations). He developed bloody stools and prolapsed rectal polyps at one year and six months, and endoscopic evaluation revealed multiple polyps in the entire intestine at two years and two months. Annual follow-up with polypectomy under general anesthesia has continued since then. During endotracheal intubation, the patient was diagnosed with difficult intubation due to macrocephaly, which limited head extension and prevented visualization of the glottic orifice. At three years old, informed consent was obtained for a single-nucleotide polymorphism array analysis, which revealed a PTEN gene mutation [46, XY, der(10)del(10)(q23.2q23.31)t(1;10)(p36.2;q23.31),t(2;7)(p13;p13)], and a definitive diagnosis of PHTS was confirmed. At four years and three months old, he visited our hospital with a one-month history of coughing and snoring. His body temperature, respiratory rate, and oxygen saturation in room air were 36.5°C, 16 breaths per minute, and 96%, respectively. Physical examination revealed grade 4 enlarged tonsils, forced breathing, and stridor. Chest radiography revealed pneumonia in the lower right lobe. He was admitted for pneumonia treatment and sleep apnea monitoring. On the second day of admission, sleep apnea was observed. Nasopharyngolaryngoscopy performed by an otolaryngologist revealed tonsillar enlargement and multiple polyps in the hypopharynx and larynx (Figures 1A-1B).

These polyps were not observed four months before polypectomy under general anesthesia, and tonsillectomy was performed for progressive acute airway obstruction. Fiberoptic nasotracheal intubation was selected for safe airway management because of intubation difficulties due to laryngeal polyps and a high risk of asphyxia without spontaneous breathing. Anesthesiologists performed intubation in the operating room, and tracheotomy was planned if intubation and mask ventilation were not possible. Numerous polyps were present in the hypopharynx and larynx. Since their removal could potentially cause airway narrowing and might develop another polyp, we decided to resect these polyps only if they were likely to cause airway obstruction. On the third day of admission, tracheal extubation was performed without complications. The tonsillar enlargement was histologically diagnosed as chronic active tonsillitis with follicular hyperplasia. Stridor did not recur, and hypopharyngeal and laryngeal polyps were not considered the cause of upper airway obstruction. Nasopharyngolaryngoscopic follow-up every six months was continued for three years after tonsillectomy without enlargement of hypopharyngeal and laryngeal polyps (Figures 1C-1D).

## Discussion

Herein, we report a pediatric case of PHTS that required fiberoptic nasotracheal intubation with spontaneous breathing due to difficulty in intubation caused by macrocephaly and multiple polyps in the hypopharynx and larynx. Difficult intubation due to papillomatous lesions in the tongue and pharynx has been reported in two adult cases of PHTS [5,6]; however, there is no previous literature discussing difficult intubation in pediatric cases of PHTS and safe airway management. This case highlights the importance of preoperative airway evaluation and planning to ensure safe airway management.

Patients with PHTS present with benign and malignant tumors in various organs [1]. All patients with PHTS are at risk of upper airway obstruction due to tonsillar hypertrophy, papillomas, lymphoid hyperplasia, and 43% of cases require tonsillectomy compared to 13.6% in the general population [7]. Anesthesia with endotracheal intubation is traditionally used for airway management during tonsillectomy [8]. For safe airway management, the risk of difficult ventilation and intubation should be considered preoperatively because brain damage due to upper airway patency can occur even at short intervals under anesthesia [4]. To successfully perform endotracheal intubation, the oral, pharyngeal, and laryngeal axes are adjusted according to the head position, and the tongue base is displaced anteriorly to visualize the pharyngeal and laryngeal structures [4]. The patient was diagnosed with difficult intubation during the first endotracheal intubation because of macrocephaly, limited cervical extension, and complicated optimal head positioning for airway alignment. In addition, the present case showed airway obstruction due to tonsillar enlargement. Preoperative nasopharyngolaryngoscopic evaluation was performed prior to tonsillectomy for other possible causes of airway obstruction, and hypopharyngeal and laryngeal polyps were discovered. These polyps are considered an additional risk factor for difficult intubation because they obstruct visualization of the glottal orifice and narrow the airway. Moreover, ventilation could be compromised if hypopharyngeal or laryngeal polyps obstruct the glottic orifice upon loss of spontaneous breathing. Considering the risks of difficult intubation and ventilation, safe airway management was discussed preoperatively with a pediatrician and an anesthesiologist, and fiberoptic nasotracheal intubation under spontaneous breathing was selected for safe airway management.

To clarify the clinical characteristics of airway obstruction and management for PHTS in childhood, we searched the PubMed database using the terms “PTEN hamartoma tumor syndromes,” “child,” and “airway obstruction” and examined the references of the retrieved articles for additional articles. Our search identified four cases, including ours, and five cases of airway obstruction have been identified (Table 1) [5,9-11].

**Table 1:** Airway obstruction for children with PHTS PHTS: PTEN hamartoma tumor syndrome

Variables	Case 1	Case 2	Case 3	Case 4	Case 5
Sex	Male	Female	Female	Not available	Male
Age at airway obstruction detection	5 years	6 years	2 years 6 months	6 months	4 years
Cause of airway obstruction	Tonsillar papilloma	Tonsillar hypertrophy	Tonsillar hypertrophy and papilloma	Hyperplastic tonsils and enlarged thymus	Tonsillar hypertrophy
Symptoms of airway obstruction	Sleep apnea	Not available	Increased tonsillar mass in a short period	Shortness of breath	Sleep apnea
Other symptoms	External hydrocephalus, macrocephaly, pigmented penile macule, developmental delay, oropharyngeal papilloma	Macrocephaly, multiple benign tumors, café-au-lait spots	Macrocephaly, lipomas, café-au-lait spots	Macrocephaly, cachexia, dysphagia, intestinal lymphoid hyperplasia	Macrocephaly, developmental delay, intestinal polyps, bilateral cryptorchidism
Treatment	Adenotonsillectomy	Tonsillectomy	Tonsillectomy	Sirolimus	Tonsillectomy
Intubation technique	Direct laryngoscopy	Not available	Not available	Not available	Fiberoptic nasotracheal intubation
Difficult intubation	No	Not available	Not available	Not available	Yes
Preoperative evaluation	No	Not available	Not available	Not available	Yes
Reference	Sharma et al. [5]	Latiff et al. [9]	Piccione et al. [10]	Schmid et al. [11]	Present case

The cause of airway obstruction in childhood was tonsillar papilloma or hyperplasia in all cases, and one case was accompanied by an enlarged thymus. Tonsillectomy was performed in four cases, and only one case was diagnosed as a difficult intubation. In addition, preoperative evaluation of the airway was not performed, except in the present case. This might be because most of the upper airway obstruction for PHTS is caused by tonsillar issues, and pharyngeal and laryngeal polyps rarely occur in childhood [12]; therefore, evaluation from the oral cavity to the larynx for the risk of difficult intubation might be underestimated.

## Conclusions

In conclusion, the symptoms of PHTS, such as macrocephaly and multiple polyps in the hypopharynx and larynx, are associated with difficulty in intubation. Although hypopharyngeal and laryngeal polyps are rare in pediatric cases of PHTS, preoperative airway assessment beyond bedside examination is essential to accurately assess intubation risk and develop safe airway management plans, potentially preventing catastrophic outcomes.
